# Do you know your PSMA-tracer? Variability in the biodistribution of different PSMA ligands and its potential impact on defining PSMA-positivity prior to PSMA-targeted therapy

**DOI:** 10.1186/s13550-024-01190-7

**Published:** 2025-01-10

**Authors:** Jan Heilinger, Katrin Sabine Roth, Henning Weis, Antonis Fink, Jasmin Weindler, Felix Dietlein, Philipp Krapf, Klaus Schomäcker, Bernd Neumaier, Markus Dietlein, Alexander Drzezga, Carsten Kobe

**Affiliations:** 1 Department of Nuclear Medicine, University Hospital of Cologne, Kerpener Straße 62, 50937 Cologne, Germany; 2 Institute of Neuroscience and Medicine, Nuclear Chemistry (INM-5), Forschungszentrum Jülich GmbH, Wilhelm-Johnen-Straße, 52428 Jülich, Germany; 3 Computational Health Informatics Program, Boston Children’s Hospital, Harvard Medical School, Boston, MA 02115 USA; 4 Institute of Radiochemistry and Experimental Molecular Imaging, University Hospital of Cologne, Kerpener Straße 62, 50937 Cologne, Germany

## Abstract

**Background:**

In clinical practice, several radiopharmaceuticals are used for PSMA-PET imaging, each with distinct biodistribution patterns. This may impact treatment decisions and outcomes, as eligibility for PSMA-directed radioligand therapy is usually assessed by comparing tumoral uptake to normal liver uptake as a reference. In this study, we aimed to compare tracer uptake intraindividually in various reference regions including liver, parotid gland and spleen as well as the respective tumor-to-background ratios (TBR) of different ^18^F-labeled PSMA ligands to today’s standard radiopharmaceutical ^68^Ga-PSMA-11 in a series of patients with biochemical recurrence of prostate cancer who underwent a dual PSMA-PET examination as part of an individualized diagnostic approach.

**Results:**

Differences in background activity among different PSMA-PET tracers lead to variations in tumor-to-background ratios (TBR). In [^18^F]F-DCFPyL-PET, TBR with the liver as the reference organ (TBR_liver_) was comparable to [^68^Ga]Ga-PSMA-11-PET, while [^18^F]F-PSMA-1007-PET and [^18^F]F-JK-PSMA-7-PET showed significantly lower values. Using the parotid gland as the reference (TBR_parotidgland_), [^18^F]F-DCFPyL-PET exhibited significantly higher values, whereas [^18^F]F-PSMA-1007-PET and [^18^F]F-JK-PSMA-7-PET were comparable. For the spleen (TBR_spleen_), [^18^F]F-JK-PSMA-7-PET was comparable, but [^18^F]F-DCFPyL-PET and [^18^F]F-PSMA-1007-PET showed significantly higher and lower values, respectively. An additional Bland-Altman analyses revealed low bias for [^18^F]F-DCFPyL-PET in TBR_parotidgland_, whereas significant differences in TBR_liver_ and TBR_spleen_ for the other tracers resulted in higher bias.

**Conclusion:**

Different PSMA-PET tracers exhibit distinct biodistribution patterns, leading to variations in tumor-to-background ratios (TBR) in reference organs such as the liver, parotid gland, and spleen. Patient selection for PSMA-directed radioligand therapy is currently based on a semiquantitative approach using the liver as a reference region in [^68^Ga]Ga-PSMA-11-PET. Thus, the use of alternative [^18^F]-labeled tracers may result in under- or overestimation of a patient’s suitability for therapy. This highlights the importance of a comprehensive understanding of the differences in tracer-specific uptake behavior for accurate decisions regarding PSMA-expression levels. However, as the patient cohort in this study is at earlier disease stages, the generalizability of these findings to later-stage patients remains unclear and requires further investigation.

## Background

Radionuclide-therapy using ^177^Lu-labeled prostate-specific membrane antigen (PSMA) ligands is an established treatment option for patients with PSMA-positive metastatic castration-resistant prostate cancer after previous medical therapy [1–3].

Due to intra- and interpatient heterogeneity of PSMA-expression, eligible patients need to be identified by PSMA-PET prior to radioligand-therapy [1, 4]. Clinical trials evaluating PSMA-targeted radioligand-therapy used [^68^Ga]Ga-PSMA-11 as the standard tracer for PSMA-PET [2, 3]. However, in clinical practice different PET-radiopharmaceuticals are employed for this purpose [5]. Indeed, from clinical perspective, most radiopharmaceuticals applied for PSMA-PET also appear to be suitable for patient selection prior to radioligand-therapy. Nevertheless, data on their comparability regarding tumoral and background tissue uptake remain limited, especially for intraindividual comparisons, as most studies published rely on matched-pair analyses to address the lack of intraindividual data [5–7]. Other studies that conduct intraindividual comparisons do exist, but often encounter limitations, such as clinically significant longer intervals between PSMA-PET scans (e.g., Popescu et al. 2024), the comparison of only background tissue uptake without considering tumoral uptake or tumor-to-background uptake ratios (e.g., Ferreira et al. 2019), or the relatively small number of evaluated patients (e.g. Pattison et al. 2022) [8–10].

Recently, we demonstrated that a semi-quantitative assessment of PSMA-expression, using the liver as a reference region, gives comparable results for [^68^Ga]Ga-PSMA-11 and [^18^F]F-DCFPyL [11]. Here, we aim to assess the intraindividual comparability of background tissue uptake as well as of tumor-to-background ratios for a wider range of ^18^F-labeled PSMA tracers.

## Methods

### Patients and scans

We retrospectively studied three groups of patients who underwent two PSMA-PET-scans in rapid succession. All patients had a biochemical recurrence of their prostate cancer and had presented for restaging in order to plan their further treatment. The standard radiopharmaceutical, [^68^Ga]Ga-PSMA-11, was always used for the first scan. Depending on availability, the additional PET-scan was carried out using either [^18^F]F-DCFPyL, [^18^F]F-PSMA-1007 or [^18^F]F-JK-PSMA-7. The selection of patients for the additional PET-scan was based on the assumption that providing further diagnostic information would significantly improve the treatment decision in each case and was carried out within an individualized approach, e.g. patients had uncertain findings at another site in addition to unambiguous metastasis-related PSMA-expression in the first scan. The same groups of patients have already been the subject of other publications focusing on different aspects [12–14]. PSMA-PET-scans were performed using a Biograph mCT 128 Flow-Edge system or 16 TruePoint system (Siemens Medical Solutions, Erlangen, Germany). Reconstruction was performed via an ordered subset expectation maximisation (OSEM) algorithm.

### Quantitative and statistical analysis

PET images were quantitatively analyzed by measuring standardized uptake values corrected for body weight (SUV) with syngo.via software (Siemens Healthineers, Erlangen, Germany).

Reference region uptake mean SUV (SUV_mean_) was measured by placing a spherical volume of interest in the right hepatic lobe, the left parotid gland, the spleen, the left gluteus muscle and the mediastinal blood pool [15]. Next, tumor lesions were identified that could be reliably detected in both [^68^Ga]Ga-PSMA-11-PET and PET using one of the alternative radiopharmaceuticals analyzed here, making them suitable for a comparative analysis of the two radiopharmaceuticals. Local disease, regional as well as distant lymph node metastasis, bone metastasis and visceral metastasis were considered for evaluation. The maximum SUV (SUV_max_) was measured within tumor lesions. Ratios of SUV_max_ of the tumor lesion as compared to SUV_mean_ of different background regions (tumor-to-background ratios [TBR]) were calculated for each PET-scan.

SPSS statistics 29.0.0.0 (IBM, Armonk, USA) and GraphPad Prism 10.1.2 (GraphPad Software, Boston, USA) were used for statistical analysis. Basic descriptive statistics were performed for patient characteristics, background activity and tumor-to-background ratios.

We compared background activity as well as tumor-to-background ratios intraindividually using the Wilcoxon matched-pair signed-rank (2 samples) test. A p-value of *p* < 0.05 was considered statistically significant.

Differences in tumor-to-background ratios were illustrated by box-plots and Bland-Altman-plots.

## Results

In total, 41 patients underwent examination with [^68^Ga]Ga-PSMA-11-PET and PET using an alternative ^18^F-labeled radiopharmaceutical. Altogether 47 comparable tumor lesions were found: 24 lesions in the [^18^F]F-DCFPyL-group, 16 lesions in the [^18^F]F-PSMA-1007-group and 7 lesions in the [^18^F]F-JK-PSMA-7-group. The distribution of metastases among all patients analyzed was as follows: 24 lymph node metastases, 18 local diseases and 5 bone metastases. Table 1 displays detailed patient characteristics and PET parameters. Table 2 showcases details for distribution of metastases in the analyzed cohorts.

**Table 1:** Patients and PET parameters

**Patient cohorts**	
*[* ^*18*^ *F]F-DCFPyL*	*n* = 13 patients
*[* ^*18*^ *F]F-PSMA-1007*	*n* = 18 patients
*[* ^*18*^ *F]F-JK-PSMA-7*	*n* = 10 patients
*Total*	*n* = 41 patients
**Patient characteristics**	
*Age*	68 years (51–86 years)
*Body weight*	87 kg (62–124 kg)
*PSA*	1.3 ng/ml (0.3–50.0 ng/ml)
**PET parameters**	
*Time between scans*	13 days (6–41 days)
*Applied activity of* ^*68*^ *Ga*	145 MBq (64–220 MBq)
*Applied activity of* ^*18*^ *F*	358 MBq (162–411 MBq)
*Time to image acquisition for* ^*68*^ *Ga*	70 min (49–129 min)
*Time to image acquisition for* ^*18*^ *F*	125 min (90–175 min)

PET positron emission tomography, n number, PSA prostate-specific antigen in blood test

**Table 2:** Lesions

[^**18**^**F]F-DCFPyL-Cohort**	***n*** ** = 24 lesions**
*Local Disease*	n = 6 lesions
*Lymphatic Metastasis*	*n* = 15 lesions
*Bone Metastasis*	*n* = 3 lesions
**[** ^**18**^ **F]F-PSMA-1007-Cohort**	***n*** ** = 16 lesions**
*Local Disease*	*n* = 9 lesions
*Lymphatic Metastasis*	*n* = 5 lesions
*Bone Metastasis*	*n* = 2 lesions
**[18 F]F-JK-PSMA-7-Cohort**	***n*** ** = 7 lesions**
*Local Disease*	*n* = 3 lesions
*Lymphatic Metastasis*	*n* = 4 lesions
*Bone Metastasis*	*n* = 0 lesions

n number

### Background tissue

In comparison to [^68^Ga]Ga-PSMA-11, liver background activity was significantly elevated in PET imaging with all evaluated ^18^F-labeled tracers. Notably, this difference in liver uptake was more pronounced for [^18^F]F-PSMA-1007 and [^18^F]F-JK-PSMA-7 than for [^18^F]F-DCFPyL. In the parotid gland, background activity relative to [^68^Ga]Ga-PSMA-11 was significantly increased with [^18^F]F-PSMA-1007 but significantly reduced with [^18^F]F-DCFPyL; no significant difference was observed with [^18^F]F-JK-PSMA-7. For spleen background activity, PET imaging with [^18^F]F-PSMA-1007 demonstrated significantly higher uptake compared to [^68^Ga]Ga-PSMA-11, while [^18^F]F-DCFPyL and [^18^F]F-JK-PSMA-7 showed significantly lower uptake. Compared to [^68^Ga]Ga-PSMA-11, blood pool background activity was significantly elevated with [^18^F]F-PSMA-1007 and [^18^F]F-JK-PSMA-7, while [^18^F]F-DCFPyL showed no significant difference. Moreover, muscle background activity was significantly higher when using [^18^F]F-PSMA-1007 compared to [^68^Ga]Ga-PSMA-11; no significant differences were observed with [^18^F]F-DCFPyL or [^18^F]F-JK-PSMA-7. A detailed summary of statistical analysis for background activity is provided in Table 3.

**Table 3:** Overview of background activities and tumor-to-background ratios for ^68^Ga-PSMA-11 and ^18^F-labeled. Radiopharmaceuticals (medians and standard deviations)

Radiopharmaceutical	[^68^Ga]Ga-PSMA-11	[^18^F]F-DCFPyL	[^18^F]F-PSMA-1007	[^18^F]F-JK-PSMA-7
**Background Activity** **[SUV**_**mean**_ **]**	(*n = 41)*	(*n = 13)*	(*n = 18)*	(*n = 10)*
*Liver*	4.6 ± 1.0	6.2 ± 1.6 ^(*)^	12.9 ± 3.2 ^(*)^	11.5 ± 2.6 ^(*)^
*Parotid gland*	15.2 ± 4.3	12.5 ± 2.5 ^(*)^	20.3 ± 5.7 ^(*)^	17.0 ± 6.8 ^(#)^
*Spleen*	*5.9* ± 2.5	*3.9* ± 2.9 ^(*)^	*11.1* ± 4.2 ^(*)^	*4.4* ± 1.3 ^(*)^
*Blood pool*	1.2 ± 0.2	1.2 ± 0.2 ^(#)^	1.4 ± 0.3 ^(*)^	1.6 ± 0.4 ^(*)^
*Muscle*	0.3 ± 0.1	0.3 ± 0.1 ^(#)^	0.5 ± 0.1 ^(*)^	0.4 ± 0.1 ^(#)^
**Tumor[SUV**_**max**_ **] to** **Background Ratio**	(*n = 47)*	(*n = 24)*	(*n = 16)*	(*n = 7)*
*Liver*	1.5 ± 2.5	1.3 ± 2.6 ^(#)^	0.6 ± 1.0 ^(*)^	0.4 ± 1.0 ^(*)^
*Parotid gland*	0.3 ± 0.7	0.7 ± 0.9 ^(*)^	0.3 ± 0.5 ^(#)^	0.2 ± 1.4 ^(#)^
*Spleen*	1.0 ± 2.1	2.6 ± 3.2 ^(*)^	0.6 ± 1.0 ^(*)^	1.3 ± 4.5 ^(#)^
*Blood pool*	5.1 ± 7.7	6.6 ± 8.0 ^(#)^	4.6 ± 6.3 ^(#)^	2.7 ± 5.5 ^(*)^
*Muscle*	17.0 ± 26.9	23.4 ± 33.9 ^(#)^	11.3 ± 20.8 ^(#)^	12.0 ± 24.4 ^(#)^

SUV standardized uptake values corrected for body weight, Background Activity [SUV_mean_] mean SUV in the specified background region, n number of lesions taken to calculate the tumor-to-background ratio, or number of patients included for background tissue measurements, Wilcoxon test [^68^Ga]Ga-PSMA-11 versus specified ^18^F-labeled radiopharmaceutical with ^(*)^ significant difference, and ^(#)^without significant difference

### Tumor-to-background ratios

Tumor-to-background ratios (TBR) with liver as reference organ (TBR_liver_) in [^18^F]F-DCFPyL-PET were comparable to those in [^68^Ga]Ga-PSMA-11-PET [11]. In contrast, TBR_liver_ values for [^18^F]F-PSMA-1007-PET and [^18^F]F-JK-PSMA-7-PET were significantly lower compared to [^68^Ga]Ga-PSMA-11-PET. When the parotid gland was used as reference organ (TBR_parotidgland_), [^18^F]F-PSMA-1007-PET as well as [^18^F]F-JK-PSMA-7-PET showed comparable values to [^68^Ga]Ga-PSMA-11-PET. However, TBR_parotidgland_ was significantly higher for [^18^F]F-DCFPyL-PET in comparison to [^68^Ga]Ga-PSMA-11-PET. When the spleen was used as reference organ (TBR_spleen_), [^18^F]F-JK-PSMA-7-PET values were comparable to [^68^Ga]Ga-PSMA-11-PET. In contrast, TBR_spleen_ was significantly higher for [^18^F]F-DCFPyL-PET and significantly lower for [^18^F]F-PSMA-1007-PET when compared to [^68^Ga]Ga-PSMA-11-PET. TBRs relative to the blood pool (TBR_BP_) for [^18^F]F-DCFPyL-PET and [^18^F]F-PSMA-1007-PET were similar to those for [^68^Ga]Ga-PSMA-11-PET, whereas TBR_BP_ was significantly lower for [^18^F]F-JK-PSMA-7-PET in comparison to [^68^Ga]Ga-PSMA-11-PET. Lastly, when using muscle as the reference tissue (TBR_muscle_), non-significant but broad variability was observed across all tracers in comparison to [^68^Ga]Ga-PSMA-11-PET.

The significant differences between [^68^Ga]Ga-PSMA-11-PET and [^18^F]F-DCFPyL-PET regarding TBR_parotidgland_, as determined by the Wilcoxon test, appear to result in a relatively low bias between the two methods, as indicated by the Bland-Altman analysis. In contrast, the significant differences observed for [^18^F]F-PSMA-1007 and [^18^F]F-JK-PSMA-7 compared to [^68^Ga]Ga-PSMA-11 regarding TBR_liver_, as well as the significant differences observed for [^18^F]F-DCFPyL-PET and [^18^F]F-PSMA-1007-PET compared to [^68^Ga]Ga-PSMA-11 regarding TBR_spleen_, result in a relatively high bias between the two methods, as reflected in the Bland-Altman analysis.

**Fig. 1:**
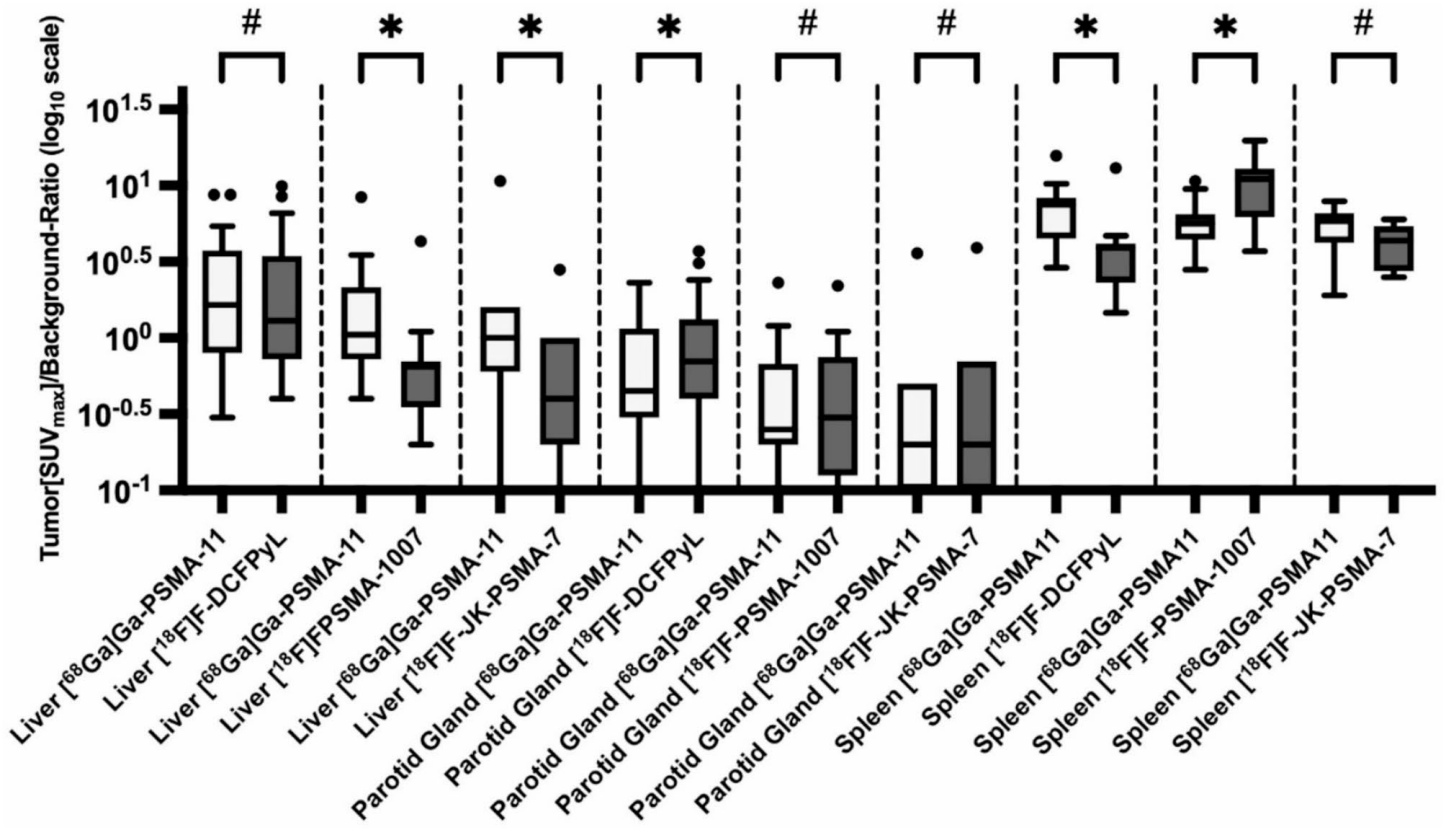
Box-plots showcasing tumor-to-background ratios in PET employing [^68^Ga]Ga-PSMA-11 as well as in PET using one of the alternative ^18^F-labeled radiopharmaceutical [^18^F]F-DCFPyL, [^18^F]F-PSMA-1007 and [^18^F]F-JK-PSMA-7 for the most clinically relevant background regions in theranostics (liver, parotid gland and spleen). This diagram is drawn with a logarithmic (log_10_) scale on the y-axis. Minimum, first quartile, median, third quartile and maximum as well as outliers are depicted. SUV standardized uptake values corrected for body weight, PET positron emission tomography, (*) significant difference in Wilcoxon test, (#) without significant difference in Wilcoxon test

**Fig. 2:**
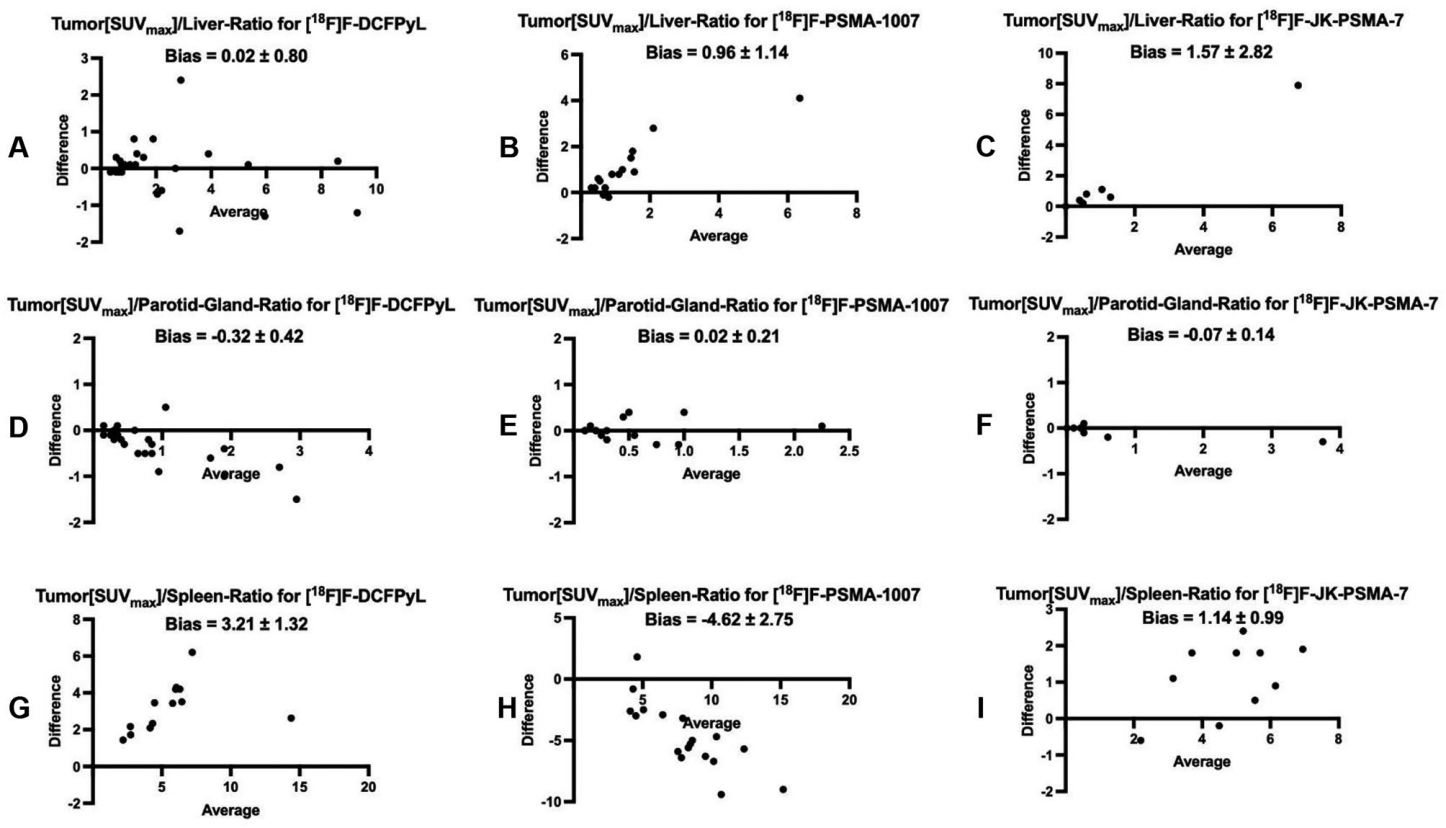
Bland-Altman plots (**A** – **I**) comparing tumor-to-background ratios in PET employing [^68^Ga]Ga-PSMA-11 as well as in PET using one of the alternative ^18^F-labeled radiopharmaceutical [^18^F]F-DCFPyL (**A**, **D**, **G**), [^18^F]F-PSMA-1007 (**B**, **E**, **H**) and [^18^F]F-JK-PSMA-7 (**C**, **F**, **I**) for the most clinically relevant background regions in theranostics (liver **A** – **C**, parotid gland **D** – **F** and spleen **G** – **I**). Bland-Altman plots in general depict the difference between two measurements as a function of the average of these measurements for each sample. In this context, bias is an indicator of the extent of the deviation in tumor-to-background ratios between the two radiopharmaceuticals. SUV standardized uptake values corrected for body weight; PET positron emission tomography

**Fig. 3:**
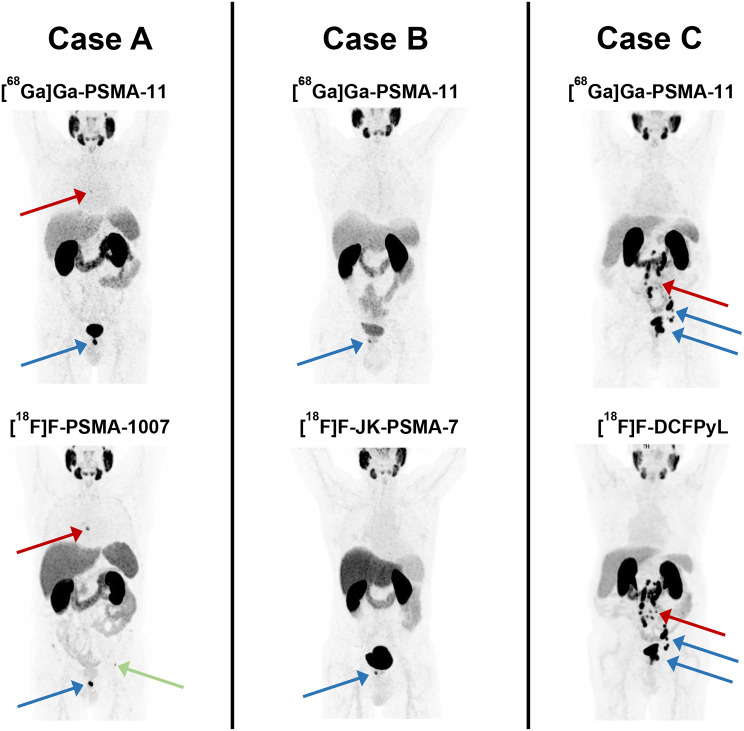
Maximum-intensity projection images from PSMA-PET scans of three patients (Case **A**, Case **B**, Case **C**) from our study are shown. Each patient received two PSMA-PET scans within a short interval: one scan employing the standard PET radiopharmaceutical [^68^Ga]Ga-PSMA-11 (top row) and a second scan using one of the analyzed ^18^F-labeled PSMA tracers (bottom row). Case **A** represents the [^18^F]F-PSMA-1007 cohort. Here a local recurrence of prostate cancer can be seen in both scans (blue arrows). Moreover, both scans reveal a small PSMA-positive lymphnode in the mediastinum, which is better shown in the ^18^F-PET showcasing the superior lesion detectability for small tumors. The green arrow marks a typical unspecific bone uptake in PET using [^18^F]F-PSMA-1007. Case **B** represents the [^18^F]F-JK-PSMA-7 cohort. Here a local recurrence of prostate cancer can be seen in both scans (blue arrow). Case **C** represents the [^18^F]F-DCFPyL cohort. This patient suffers from a local recurrence of prostate cancer as well as an extensive lymphonodal metastasis in the retroperitoneum (blue arrows) shown on both scans. Nevertheless, some of the retroperitoneal metastasis can be seen better in the ^18^F-PET showcasing the superior lesion detectability for small tumors. PSMA prostate specific membrane antigen; PET positron emission tomography

Details of the statistical analyses are provided in Table 3, while the comparison of tumor-to-background ratios most relevant for theranostics between the standard radiopharmaceutical [^68^Ga]Ga-PSMA-11 and competing ^18^F-labeled PSMA-PET tracers is visualized using box plots (Fig. 1) and Bland-Altman plots (Fig. 2). Figure 3 shows an intraindividual image comparison of PET scans using the ^18^F-labeled radiopharmaceuticals and [^68^Ga]Ga-PSMA-11.

## Discussion

The following findings emerge from our analysis:

- Tumor-to-background ratios (TBR) with liver as reference organ (TBR_liver_) in [^18^F]F-DCFPyL-PET were comparable to that in [^68^Ga]Ga-PSMA-11-PET [11]. In contrast, TBR_liver_ values for [^18^F]F-PSMA-1007-PET and [^18^F]F-JK-PSMA-7-PET were significantly lower compared to [^68^Ga]Ga-PSMA-11-PET (high bias in Bland-Altman-Analysis).
- When the parotid gland was used as reference organ (TBR_parotidgland_), [^18^F]F-PSMA-1007-PET as well as [^18^F]F-JK-PSMA-7-PET showed comparable values to [^68^Ga]Ga-PSMA-11-PET. However, TBR_parotidgland_ was significantly higher for [^18^F]F-DCFPyL-PET in comparison to [^68^Ga]Ga-PSMA-11-PET (low bias in Bland-Altman-Analysis).
- When the spleen was used as reference organ (TBR_spleen_), [^18^F]F-JK-PSMA-7-PET values were comparable to [^68^Ga]Ga-PSMA-11-PET. In contrast, TBR_spleen_ was significantly higher for [^18^F]F-DCFPyL-PET and significantly lower for [^18^F]F-PSMA-1007-PET compared to [^68^Ga]Ga-PSMA-11-PET (high bias in Bland-Altman-Analysis).

These findings are of clinical importance since PSMA-positivity of tumor manifestations is predictive for the efficacy of PSMA-directed radioligand-therapy and is usually defined as tumoral PSMA-expression above the hepatic background activity using [^68^Ga]Ga-PSMA-11 [2, 16]. In a clinical context this means that the higher liver uptake of [^18^F]F-JK-PSMA-7 or [^18^F]F-PSMA-1007 may cause patients to be missed, who might have benefited from a radioligand-therapy, through underestimation of their tumoral uptake [17]. Nevertheless, one can be sure that tumoral uptake above liver background imaged with these radiopharmaceuticals is indicative of a patient eligible for radioligand therapy, as liver uptake of [^18^F]JK-PSMA-7 or [^18^F]F-PSMA-1007 is significantly higher than that observed with [^68^Ga]Ga-PSMA-11 [17]. The significantly higher liver uptake for [^18^F]F-PSMA-1007 was also found by Popescu et al. (2024) [8]. In the case of uncertainties, an alternative reference region could be an option. Our results suggest that the parotid gland presents a relatively constant reference region across the radiopharmaceuticals analyzed here, with the exception of [^18^F]F-DCFPyL, as the significantly higher TBR_parotidgland_ of [^18^F]F-DCFPyL could lead to an overestimation of PSMA-expression. While the Wilcoxon test identified statistically significant differences in the comparison of TBR_parotidgland_ in [^18^F]F-DCFPyL-PET and [^68^Ga]Ga-PSMA-11-PET, the low bias observed in the Bland-Altman analysis suggests that these differences may be of limited practical relevance. Overall, the parotid gland demonstrates a largely consistent reference region across the tracers analyzed, although its uptake is in general consistently higher compared to liver uptake in [^68^Ga]Ga-PSMA-11-PET, which has to be taken into account when using it as a cut-off for decision on radioligand-therapy. Another commonly discussed reference region is the spleen [18]. Interestingly, TBR_spleen_ showed no significant differences in the comparison between [^68^Ga]Ga-PSMA-11-PET and [^18^F]F-JK-PSMA-7-PET. Given the fact that spleen uptake using [^18^F]F-JK-PSMA-7 appears to be roughly in the range of liver uptake obtained with [^68^Ga]Ga-PSMA-11, the spleen has the potential to serve as an alternative reference region for semiquantitative assessment of pretherapeutic PSMA-positivity in [^18^F]JK-PSMA-7-PET, where liver uptake is significantly higher than in [^68^Ga]Ga-PSMA-11-PET. Nonetheless, we observed significant differences with a high bias in the Bland-Altman analysis for TBR_spleen_ in the comparison of [^68^Ga]Ga-PSMA-11 to [^18^F]F-DCFPyL or [^18^F]F-PSMA-1007. In addition to that, a significantly higher spleen uptake for [^18^F]F-PSMA-1007 was also shown previously by Popescu et al. (2024) [8]. All in all, when using [^18^F]F-DCFPyL or [^18^F]F-PSMA-1007, the spleen cannot be recommended as a reliable alternative reference region.

As a future perspective, the comparability of TBRs across different tracers could be improved by the introduction of conversion factors to account for variations in background activity. Such a factor has already been proposed by Popescu et al. (2024) for liver background uptake between [^68^Ga]Ga-PSMA-11-PET and [^18^F]F-PSMA-1007-PET and appears to be applicable to our data [8].

Our present study has some limitations. First, our observations are based on a highly select group of patients with biochemical recurrence of prostate cancer. We had therefore most likely focused on patients suffering from small tumor volumes, who had not undergone systemic treatment such as chemotherapy or androgen receptor pathway inhibitors. Moreover, due to the earlier stage of disease, bone as well as visceral metastases are under-represented in the analyzed cohorts but are common findings in patients qualifying for radioligand-therapy. As all of these aspects may affect reference organ and / or tumor uptake of the analyzed PSMA-tracers it remains unclear whether our results are transferable to patients who are actually selected for radioligand-therapy. Furthermore, it has to be taken into account that the markedly longer time to image acquisition for ^18^F-labeled PSMA-tracers in this study can be seen as another limitation, as this may have an effect on tumor and background activity measurements as well as tumor-to-background ratios. However, the possibility of making a direct comparison within the same patients does represent a strength of the current study despite the small sample size. In general, it will be difficult to obtain dual PET-data for direct comparison and most previous studies have used matched-pair analyses to compensate for the lack of data on intraindividual comparisons [5–7]. Other studies that conduct intraindividual comparisons do exist, but often encounter limitations, such as clinically significant longer intervals between PSMA-PET scans (e.g., Popescu et al. 2024), the comparison of background tissue uptake alone without considering tumoral uptake or tumor-to-background uptake ratios (e.g., Ferreira et al. 2019), or the relatively small number of evaluated patients (e.g. Pattison et al. 2022) [8–10]. All in all, it seems reasonable to assume that a reliable impression of the general tracer distribution behavior with regard to tumor-to-background ratios can be drawn from the current sample. Therefore, despite its limitations, this study does help to provide further information on the comparability of these tracers.

## Conclusion

Different PSMA-PET tracers exhibit distinct biodistribution patterns, resulting in variations in tumor-to-background ratios (TBR) with reference organs such as the liver, parotid gland and spleen. Since patient selection for PSMA-directed radioligand therapy currently relies on a semiquantitative approach using the liver as a reference organ in [^68^Ga]Ga-PSMA-11-PET, the use of alternative [^18^F]-labelled tracers may lead to differences in TBR, potentially underestimating or overestimating a patient’s suitability for treatment. Despite these variations, we consider all the tracers analyzed here to be appropriate for pretherapeutic evaluation of PSMA-expression. However, caution should be exercised when establishing semi-quantitative reference cut-offs, and a thorough understanding of tracer-specific uptake behavior is crucial for making accurate pretherapeutic decisions. Nevertheless, as the patient cohort in this study was at earlier disease stages, further investigation in later-stage patients is necessary to verify these findings.

## Data Availability

The datasets generated and analyzed during this study are available from the corresponding author on reasonable request.
